# Partial Small Bowel Obstruction and Volvulus Due to B-cell Lymphoma in a Common Variable Immunodeficiency Patient

**DOI:** 10.7759/cureus.47269

**Published:** 2023-10-18

**Authors:** Reshmi Adupa, Harika Dadigiri, Darshan Gandhi

**Affiliations:** 1 Internal Medicine, New York Medical College at St. Mary's General Hospital, Passaic, USA; 2 Internal Medicine, New York Medical College at St. Clare's Health, Denville, USA; 3 Internal Medicine, Sri Venkateswaraa Medical College, Tirupati, IND

**Keywords:** immunodeficiency, mesenteric volvulus, small-bowel obstruction, b-cell lymphoma, common variable immunodeficiency deficiency

## Abstract

This case report presents a 43-year-old female with a history of common variable immunodeficiency (CVID) and a recent diagnosis of mesenteric volvulus. The patient presented with symptoms of partial small bowel obstruction and was diagnosed with obstruction and mesenteric volvulus primarily affecting the proximal jejunum. During the exploratory laparotomy, a probable polyposis syndrome and a possible adenocarcinoma of the small bowel were identified. Pathological examination confirmed the presence of B-cell lymphoma in the proximal jejunum. The patient underwent treatment with rituximab, cyclophosphamide, doxorubicin, vincristine, and prednisolone (RCHOP) chemotherapy and showed improvement in symptoms. The case highlights the increased risk of malignancies, particularly lymphomas, in individuals with CVID and the challenges in diagnosing and treating lymphoid neoplasms in this population.

## Introduction

The prevalence of primary immunodeficiencies and immunological dysregulatory diseases is 41.4 or 50.5 per 100,000 people, respectively. According to research conducted in the United States and Europe, common variable immunodeficiency (CVID) is the most prevalent adult humoral immunodeficiency [1]. Recurrent sinopulmonary infections, autoimmune diseases, and granulomatous illness with a higher risk of malignancy are all characteristics of CVID [2]. Malignancy is prevalent in people with primary immunodeficiency and is the second-largest cause of death in both children and adults, after infection [1]. Extranodal non-Hodgkin's lymphoma (NHL) is most commonly seen in the GI system, and its frequency has grown recently. The stomach (50-60%) and small intestine (20-30%) are the two major locations that are used the most frequently [3]. The small intestine is the location of intestinal lymphoma that occurs most frequently; large bowel and rectal lymphomas are less frequent. Diffuse large B-cell lymphoma (DLBCL) makes up the bulk of primary intestinal B-cell lymphomas; however, there are additional histologic subtypes that can occur, such as intestinal mucosa-associated lymphoid tissue lymphoma, follicular lymphoma, mantle cell lymphoma, etc. [4]. This case report discusses a patient with CVID who presented with symptoms of small bowel obstruction and was subsequently diagnosed with B-cell lymphoma in the proximal jejunum. The report highlights the complexities of diagnosing and treating lymphoid neoplasms in CVID patients and the importance of a multidisciplinary approach.

## Case presentation

A 43-year-old female with a past medical history of CVID and receiving intravenous immunoglobulin of 15 grams once a week has a surgical history that includes a prior cesarean section and tubal ligation presented with complaints of abdominal pain and abdominal distension. The patient had been experiencing chronic diarrhea for four months, which was initially attributed to a diagnosis of Giardia. However, her symptoms worsened over the past 24 to 48 hours, accompanied by sudden abdominal pain and distention. The patient denied nausea, vomiting, significant weight loss, difficulty swallowing, and blood in stools. In terms of vital signs, the patient's blood pressure ranged from 87/45 to 100/55 mmHg, pulse rate ranged from 90 to 97 beats per minute, respiratory rate ranged from 20 to 22 breaths per minute, and weight was recorded at 51.8 kg over a 24-hour period.

The patient denied any history of smoking, use of smokeless tobacco, alcohol consumption, or drug use. However, her family history revealed a case of breast cancer in her mother. Laboratory tests showed potassium -3.1 (normal (n)=3.7-5.2 mEq/L), mild anemia (hemoglobin: 11.1 g/L, n=11.6-15 g/L), aspartate transaminase -52 U/L (n=8-33 U/L), alanine transaminase -49 U/L (n=4-36 U/L), alkaline phosphatase -190 U/L (20-130 U/L), total protein -4.2 g/dL (n=6-8.3 g/dL), and albumin -2 g/dL (3-4-5.4 g/dL). CA 125 elevated with the level of 40 U/mL (n=0-35 U/mL). Tumor markers, serum AFP, CA 19-9, and CEA, are negative. Stool cultures were negative for Helicobacter pylori, Campylobacter jejuni, Escherichia coli 0157, Salmonella, Shigella, Yersinia, Aeromonas, Plesiomonas, and ova and parasites.

**Figure 1:**
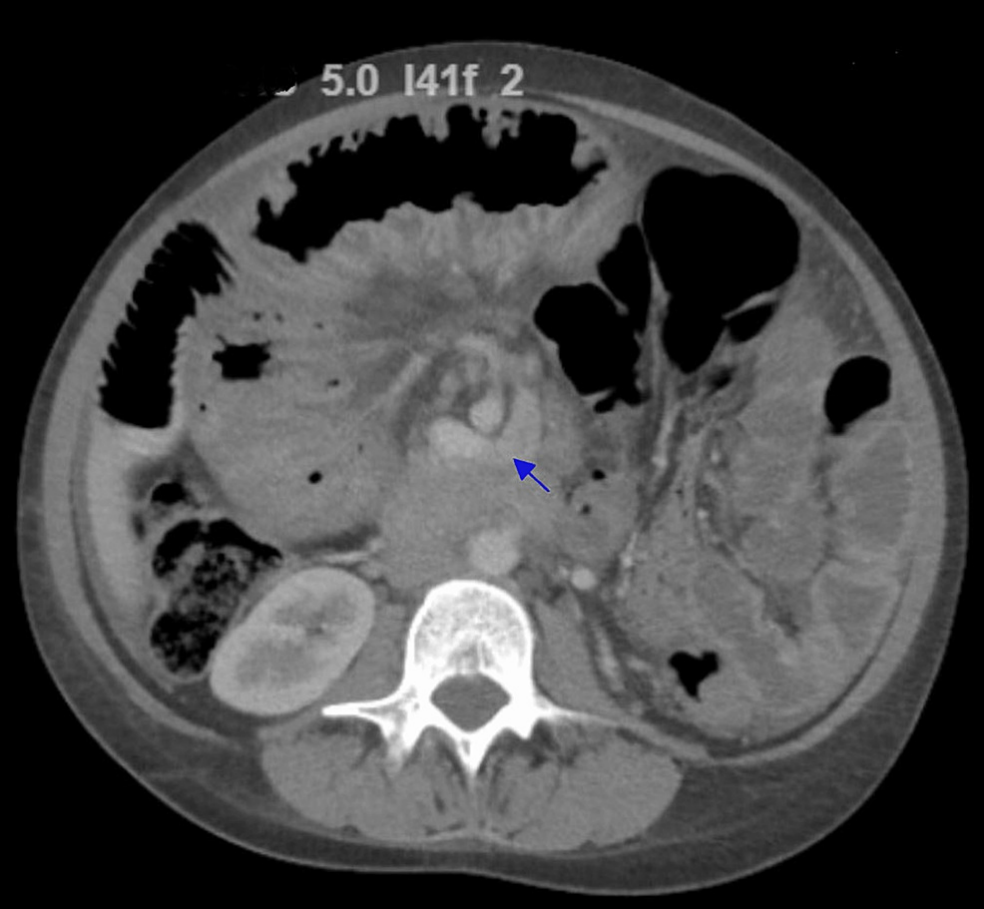
CT of the abdomen with contrast CT of the abdomen showing swirling of the mesentery

The CT scan showed swirling of the mesentery, confirming the presence of mesenteric volvulus and dilated proximal small bowel loops (Figure 1). The patient was diagnosed with obstruction and mesenteric volvulus, primarily affecting the proximal jejunum. Urgent laparotomy was performed, involving adhesion lysis, partial small bowel resection, and reanastomosis.

The surgical report indicated a probable polyposis syndrome and adenocarcinoma of the small bowel. Pathological examination of the resected tissue indicated B-cell lymphoma with Ki-67 close to 90% in the proximal jejunum, along with intramucosal reactive lymphoid follicles. Further classification was requested through FISH studies, which showed no evidence of MYC, BCL2-IGH, or BCL 6 rearrangements. The bone marrow was negative for DLBCL.

The patient's hospital course involved postoperative recovery after a partial small bowel obstruction due to mesenteric volvulus. A seven-day course of IV piperacillin and tazobactam was administered for broad-spectrum antibiotic coverage to prevent infection in the postoperative period. The patient showed gradual improvement in her abdominal pain and distention following the surgery.

Regarding the treatment for B-cell lymphoma, the patient completed six cycles of rituximab 600 mg, cyclophosphamide 1,155 mg, doxorubicin 77 mg, vincristine 2 mg, and prednisolone oral 100 mg (RCHOP) and repeated her completion PET-CT which showed 1.9 x 0.9 cm dense central mesenteric node again visualized with the maximum standardized uptake value (SUV max) of 4.0 and Deauville score of 4. The previous showed 2.0 x 1.0 cm with an SUV max of 4.1 in the left mesentery. In addition to antibiotic therapy, the patient received intravenous fluids to maintain hydration and electrolyte balance. Pain management was achieved using analgesics, primarily focusing on opioid-sparing medications to minimize the risk of postoperative ileus.

## Discussion

While selective IgA deficiency is the most common primary immunodeficiency, it is often asymptomatic. On the other hand, CVID is the most clinically relevant primary immunodeficiency. The well-established definition of CVID includes three essential features: hypogammaglobulinemia affecting two or more immunoglobulin isotypes (low IgG, IgA, or IgM), recurrent infections of the sinopulmonary system, and impaired functional antibody responses [5]. Research has indicated that mutations in genes such as H159Y, LRBA, NFKB1, PIK3CD variants, IKZF1, or Ikaros have been associated with the onset of lymphoma in individuals with CVID [6]. The mainstay of treatment for CVID is the administration of antibody replacement therapy, which can be delivered intravenously or subcutaneously. The initial dose typically ranges from 400 to 600 mg/kg of gamma globulin per month. Individuals diagnosed with CVID face a heightened risk of developing malignancies, with occurrences observed in up to 15% of patients. Notably, there is an elevated prevalence of NHL and gastric carcinoma in individuals with CVID, contributing significantly to morbidity and mortality. These lymphomas tend to be more prevalent among individuals aged between their fourth and seventh decades of life [7]. The estimated risk of developing lymphoma in individuals with CVID ranges from 1.4% to 7%. Approximately 2-8% of individuals diagnosed with CVID also develop NHL [8]. Lymphoma exhibits heterogeneity and is influenced by various factors such as compromised immunosurveillance, persistent infections, and genetic predisposition [9].

The underlying mechanisms contributing to lymphoma development in CVID remain incompletely understood, but factors such as high IgM levels, late-onset combined immunodeficiency phenotype, and polyclonal lymphoproliferative disease have been associated with an elevated risk [10]. Additionally, genetic predisposition may play a role, with specific germline variants potentially influencing somatic alterations in lymphoma [11]. The occurrence of malignancies in the small intestine is on the rise. The five-year survival rates are generally low, with an overall rate of 54% [12]. The World Health Organization categorizes lymphoid neoplasms into groups, including precursor lymphoid neoplasms, mature B-cell neoplasms, mature T-cell and natural killer cell neoplasms, and NHL. Extranodal Hodgkin's lymphoma is exceedingly rare. B-cell lymphomas predominate in extranodal GI lymphomas (80%), responding well to chemotherapy with a favorable prognosis. GI lymphomas account for 1-4% of GI malignancies, 10-15% of NHLs, and 30-40% of extranodal NHLs, with the GI tract as the most common extranodal lymphoma site [13]. Clinical gastroenterologists are confronted with the complexity of diverse tumor presentations in these cases. Symptoms can range from nonspecific issues like dyspepsia or bloating to more specific manifestations such as abdominal pain, nausea, vomiting, GI bleeding, diarrhea, weight loss, or bowel obstruction. Patients may present with a combination of these symptoms, including fever, abdominal pain, diarrhea, passage of bloody stools (hematochezia), and weight loss [13]. Lymphoma often has nonspecific symptoms like fever, weight loss, and night sweats occurring in less than 12% of cases. Acute abdominal pain leads to hospitalization in 30- 50% of patients, and about 25% of these cases involve GI perforation. Obstruction or perforation, though rare, significantly raises mortality risk due to complications like sepsis, multi-organ failure, prolonged hospital stays, impaired healing, and chemotherapy delays [14]. Bowel obstruction is a significant contributor to morbidity and mortality, causing nearly 30,000 deaths annually. The primary causes in 90% of small bowel obstruction cases are adhesions, hernias, and neoplasms. Treatment for small intestine obstruction induced by small bowel tumors, including adenocarcinoma, neuroendocrine tumors, GI stromal tumors, and lymphomas, typically involves excision and anastomosis [15]. PET-CT is valuable for initially staging lymphoma accurately. However, its ability to assess treatment response is constrained by potential false positives due to concurrent inflammation or infection and false negatives, as it may not detect microscopic disease. These limitations are evident in the variable outcomes observed when using the Deauville score visual assessment to gauge early treatment response [16]. Surgical treatment was historically the primary approach for managing primary GI lymphoma (PGIL), often involving radical resection, and palliative resection for extensive cases. However, the high sensitivity of lymphoma to chemotherapy has shifted the treatment paradigm toward non-surgical methods. A prospective study found that adding surgery to chemotherapy did not significantly enhance the 10-year survival rate of PGIL compared to chemotherapy alone. Consequently, a substantial portion of patients (54.5%) now receive non-surgical treatments like CHOP or RCHOP (rituximab, cyclophosphamide, doxorubicin, vincristine, and prednisolone) chemotherapy regimens, with the introduction of rituximab, a CD20-targeting monoclonal antibody, improving overall survival by eliminating both normal and malignant B-cells expressing CD20 [17].

## Conclusions

CVID is a clinically relevant primary immunodeficiency associated with hypogammaglobulinemia and recurrent infections. Individuals with CVID are at an increased risk of developing malignancies, including lymphomas. The mechanisms underlying lymphoma development in CVID are not fully understood. Primary GI lymphomas, particularly B-cell lymphomas, are more prevalent in CVID patients and require careful diagnosis due to overlapping features with lymphoid hyperplasia. Further research is needed to better understand the underlying mechanism and identify specific risk factors that contribute to lymphoma development in CVID patients by focusing on investigating the intricate interplay between immune dysregulation, genetic predisposition, and the microenvironment to optimize management strategies for lymphomas in CVID patients. Prompt recognition and appropriate treatment, such as chemotherapy regimens like RCHOP, are crucial for improving outcomes in CVID patients with lymphomas.
